# Medical 3D Printing Using Desktop Inverted Vat Photopolymerization: Background, Clinical Applications, and Challenges

**DOI:** 10.3390/bioengineering10070782

**Published:** 2023-06-30

**Authors:** Parimal Patel, Kashish Dhal, Rajul Gupta, Karthik Tappa, Frank J. Rybicki, Prashanth Ravi

**Affiliations:** 1 Department of Mechanical & Aerospace Engineering, University of Texas at Arlington, Arlington, TX 76019, USA; parimalthakorbh.patel@mavs.uta.edu (P.P.); kashish.dhal@mavs.uta.edu (K.D.); 2 Department of Orthopedic Surgery, University of Cincinnati, Cincinnati, OH 45219, USA; gupta2rl@ucmail.uc.edu; 3 Department of Breast Imaging, Division of Diagnostic Imaging, The University of Texas MD Anderson Cancer Center, Houston, TX 77030, USA; kktappa@mdanderson.org; 4 Department of Radiology, University of Cincinnati, Cincinnati, OH 45219, USA; rybickfk@ucmail.uc.edu

**Keywords:** medical 3D printing, desktop vat photopolymerization, anatomical models, surgical guides, medical devices, accuracy

## Abstract

Medical 3D printing is a complex, highly interdisciplinary, and revolutionary technology that is positively transforming the care of patients. The technology is being increasingly adopted at the Point of Care (PoC) as a consequence of the strong value offered to medical practitioners. One of the key technologies within the medical 3D printing portfolio enabling this transition is desktop inverted Vat Photopolymerization (VP) owing to its accessibility, high quality, and versatility of materials. Several reports in the peer-reviewed literature have detailed the medical impact of 3D printing technologies as a whole. This review focuses on the multitude of clinical applications of desktop inverted VP 3D printing which have grown substantially in the last decade. The principles, advantages, and challenges of this technology are reviewed from a medical standpoint. This review serves as a primer for the continually growing exciting applications of desktop-inverted VP 3D printing in healthcare.

## 1. Introduction

Medical three-dimensional (3D) printing of anatomic models, guides, implants, and other devices is transforming medical practice and patient care [1]. Increasingly, with the lowering of costs associated with medical 3D printing technologies and in anticipation of future reimbursement [2], 3D printing is moving into healthcare centers and this is now being referred to as Point-of-Care (PoC) 3D printing [3]. The United States (US) Food and Drug Administration (FDA) published a discussion paper soliciting comments from the community to develop future regulations for 3D printing medical devices at the PoC [4]. Anatomic models fabricated for diverse clinical scenarios at the PoC using 3D printing have shown high clinical utility and the comprehensive costs for fabricating these models in-house as well as if outsourced to industry have been benchmarked [5]. Although the terminology “3D printing” has gained popularity, it is in some sense a misnomer because 3D printing is really a manufacturing technology that can be used to fabricate physical parts, oftentimes with complex geometries that cannot be achieved using other manufacturing methods. Traditionally, manufacturing was subtractive where one started off with a larger block of material that was gradually chipped away either manually or using machines to achieve the desired part. This was completely upended by 3D printing due to the “additive” nature of the process where material is controllably added in a layer-by-layer fashion to achieve the desired part. Within “3D printing” or “additive manufacturing” there are seven different technologies as defined by the ASTM: Material Extrusion (MEX), Vat Photopolymerization (VP), Powder Bed Fusion (PBF), Binder Jetting (BJT), Sheet Lamination (SL), Material Jetting (MJT), and Directed Energy Deposition (DED) [6]. Collectively these seven technologies allow the fabrication of materials using polymers, metals, composites, and ceramics.

Although 3D printing technologies have been around for over 35 years, the printers were large and prohibitively expensive for most users except those in industries such as automotive and aerospace. Furthermore, the technology was primarily used for prototyping and not to manufacture functional end-use parts. With the expiration of key patents in 3D printing after the turn of the millennium and the progress in underlying technologies including motion control and optics, the bulky industrial VP 3D printing technology was miniaturized and translated into the desktop space by inverting the technology from the conventional top-down approach to the new bottom-up style. This was an important development that dramatically lowered the cost-barrier to VP 3D printing from over $100,000 to around $5000. At present, there are only Current Procedural Terminology (CPT) category III codes for 3D printed anatomic models and 3D printed anatomic guides [2] which do not guarantee reimbursement. Therefore, a low entry cost-barrier to 3D printing is important to enable wider adoption of this technology within hospitals, particularly since a PoC 3D printing laboratory will largely be an expense to hospitals till CPT I reimbursement codes become available for medical 3D printing, despite the fact that 3D printing has been shown to reduce operative room costs [5]. Desktop inverted VP 3D printing systems have proliferated across multiple industries including healthcare due to their lower cost, smaller footprint, versatility in materials, and ease of maintenance. Furthermore, this technology enables the fabrication of models with high accuracy and surface quality, characteristics particularly desirable in the medical domain.

While there are several literature surveys regarding medical 3D printing, there are no detailed reviews of clinical desktop VP 3D printing. A recent review of polymer 3D printing, including MEX, VP, and PBF technologies, focused on research advances related to the technical aspects of materials, processes, and design strategies for medical applications including tissue-engineered scaffolds [7]. A 2020 review focused on all types of 3D printing technologies in healthcare and medicine including applications in pharmaceuticals, dentistry, pre-surgical planning, and tissue engineering [8]. A 2019 review described the current and future applications of 3D printing technologies in customized medical devices, drug-eluting implants, tablets, dentistry, surgeries, and bioprinting of patient-specific organs [9]. A 2018 review delved into the pharmaceutical and biomedical application of 3D printing technologies with a focus on MEX-based methods [10]. A different 2018 review focused solely on the endodontic applications of 3D printing technologies [11]. Another 2018 article reviewed recent developments in 3D printing technologies in medical applications [12]. A 2016 review highlighted the medical applications of 3D printing revolutionizing surgeries, prostheses, implants, dentistry, and bioprinting [13]. A 2015 article reviewed plastic surgery applications of 3D printing including templates for facial transplantation surgery to craniofacial implants for optimizing post-operative aesthetics [14]. One of the oldest and at that time comprehensive reviews of the medical applications of 3D printing based on imaging data was published in 2010 [15]. A 2016 review summarized the literature on patient-specific surgical 3D printing applications with a focus on the reported clinical and economic outcomes [16]. A 2015 review provided a comprehensive insight into the technical and clinical aspects of the medical 3D printing workflow and example clinical applications [2].

The purpose of this review is to detail the typical medical 3D printing workflow from source images to the final model, describe the principles of desktop inverted VP 3D printing, and discuss the advantages of the technology compared to other technologies. This review also highlights the medical applications of desktop VP 3D printing, and the major challenges of the technology as they relate to medicine. This review is not structured as a systematic review, but rather provides a comprehensive assessment of the role played by desktop VP 3D printing in medicine with the end goal of addressing that very literature gap. The authors recognize that stereolithography (SLA) is a type of VP 3D printing, with the former term being a trade name and the latter being the generic description of the technology. Only the term VP is used in this review and more specifically, the desktop inverted VP 3D printing is used since that is the focus of this manuscript.

## 2. Medical 3D Printing Workflow

**Figure 1:**
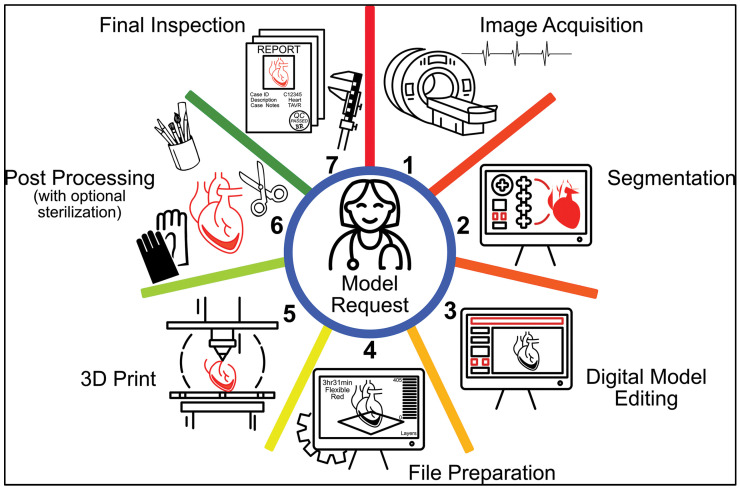
Steps involved in the complex and multidisciplinary medical 3D printing workflow. Radiologists, 3D printing engineers, and clinicians are the key stakeholders involved in this process, and close communication between them is a crucial piece of the process. The wheel of medical 3D printing is first set in motion when a physician requests a model. Once the order is placed, the first step involves proper image acquisition (1) which is then followed by segmentation (2) of the anatomic structures of interest using specialized software. The surface mesh file made from the segmented model is digitally edited (3). This editing can involve several operations such as smoothing, labeling, addition of geometric features etc. The finalized model is then prepared for 3D printing (4) in the appropriate software. Post 3D printing (5), the model is post-processed (6) as needed based on the technology used to 3D print. The finished model is then inspected (7) to ensure quality and safety. Reprinted/adapted with permission from Ref. [18]. Copyright 2022, RSNA.

Medical 3D printing involves a series of discrete steps (Figure 1). The workflow is complex, effort-intensive, and requires cross-disciplinary expertise to ensure that the anatomical models produced are of good quality and safe to be used in patient care. This section of the manuscript will go over each of these steps. The key stakeholders at the center include the 3D printing engineer, the radiologist, and a physician provider who performs the medical service of 3D printing in a Health Care Facility (HCF). Because 3D printing is a medical service in the United States [17], it is performed by a provider. In many HCFs, the 3D printing provider is a radiologist. The clinician has the medical expertise to assess in which cases and how he can truly benefit from the anatomic model as part of his planning. The 3D printing engineer is the technical expert who can perform the segmentation, computer-aided design (CAD), and 3D printing. The radiologist is a provider who is clinically responsible for the service and is the reference standard for image segmentation as well as the overall quality control and clinical utilization of the 3D printed parts. Proper communication between all these stakeholders is paramount to ensure that the models produced address the medical challenge effectively while ensuring the safety of the patient.

### 2.1. Image Acquisition

**Figure 2:**
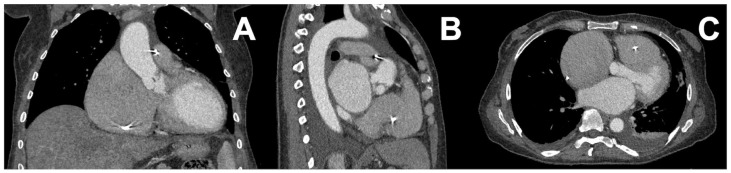
(**A**) Coronal, (**B**) Sagittal reformatted images from a dedicated cardiac CT acquisition. (**C**) Representative axial slice through the heart. These data are the foundation of planning an endoscopic minimally invasive valve repair. This patient underwent triple (aortic, mitral, tricuspid) valve repair/replacement surgery. Using a thin slice image set (<1.25 mm slice thickness) the regions of interest can be segmented for 3D printing and other virtual planning using CAD.

All patient-specific medical VP 3D printing uses images and the quality of the images greatly impacts the quality of the final 3D printed model, because the images serve as the input dataset from which the anatomical structures of interest are extracted and processed for 3D printing. Computed Tomography (CT) is a widely accepted scanning method since it provides good spatial resolution, contrast, and signal-to-noise ratio to segment regions of interest that can then be 3D printed [19]. Such a scan is depicted in Figure 2 for planning an endoscopic minimally invasive cardiac valve repair/replacement surgery. Magnetic Resonance Imaging (MRI) is also widely used [15]. Other techniques including Positron Emission Tomography (PET), Ultrasonography, Single Photon Emission Computed Tomography (SPECT), and Cone Beam Computed Tomography (CBCT) are also used standalone or in conjunction with image sets obtained from different modalities.

Based on the current capabilities of those scanning methods, images with contrast and signal-to-noise properties adequate for accurate segmentation can be obtained at sub-millimeter spatial resolution in all three planes, making volumetric images reconstructed with a slice thickness of 1 mm suitable for medical 3D printing. The data acquired from the scanners are recorded in DICOM (Digital Imaging and Communications in Medicine) format which stores both the pixel data along with other metadata in the header that includes medical as well as imaging information. Some images are post-processed to remove any artifacts or errors so that they are not carried over into the printed models [20,21]. Specific reconstruction kernels such as those for bone may be applied for better visualization of the tissue of interest. A slice interval of 1.25 mm or lower is required in the source images for accurate 3D printing of anatomical structures. Proper contrast opacification and a high signal-to-noise ratio are necessary for accurate 3D printing.

### 2.2. Segmentation

**Figure 3:**
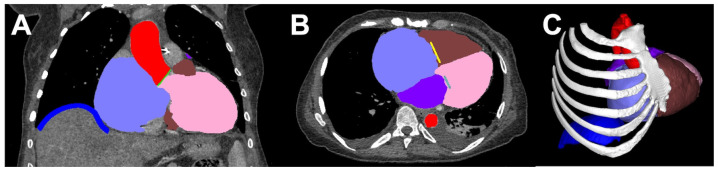
(**A**) Coronal view showing the segmented voxel masks overlaid on the CT images, right hemidiaphragm (dark blue), right atrium (lavender), aorta (red), left ventricle (pink), aortic valve (green). (**B**) Axial view showing the segmented masks overlaid on the CT images, left atrium (purple), right ventricle (brown), tricuspid valve (yellow), mitral valve (cyan). (**C**) The 3D preview of the segmented masks before creation of a surface mesh for further manipulation in CAD and subsequent 3D printing.

Segmentation can be defined as the delineation or extraction of specific anatomical structures or tissues in the form of regions of interest (voxel datasets) from the image data (Figure 3). A DICOM file is either manually or automatically processed using software to remove surrounding structures [22,23]. Often, the segmentation is performed using semi-automatic techniques. Proper training in the software, processes, and anatomy is needed to avoid introducing any error during segmentation as it is easy to misinterpret a feature inside the scan with poor knowledge. In the majority of cases, the segmentation process, compared to other steps in the entire medical 3D printing workflow, is the most labor-intensive and time-consuming. In fact, given the high accuracy of 3D printers in recent times and the complexities involved in image segmentation, this step often contributes to the majority of dimensional errors in the final model. The contours defined through segmentation are normally transformed into Standard Tessellation Language (STL) files that are composed of 3D triangular meshes which later can be used for 3D printing. Additional file formats such as Wavefront OBJ (OBJ), Virtual Reality Modeling Language (VRML) and extensible 3D (X3D) are available which offer additional features like color, and texture. When selecting the file format, mesh criteria should be chosen for fine detail and optimum file size that does not overburden the processor [1]. The mesh file is then transferred to computer-aided design (CAD) software for further processing.

### 2.3. Computer-Aided Design (CAD) or Digital Model Editing

**Figure 4:**
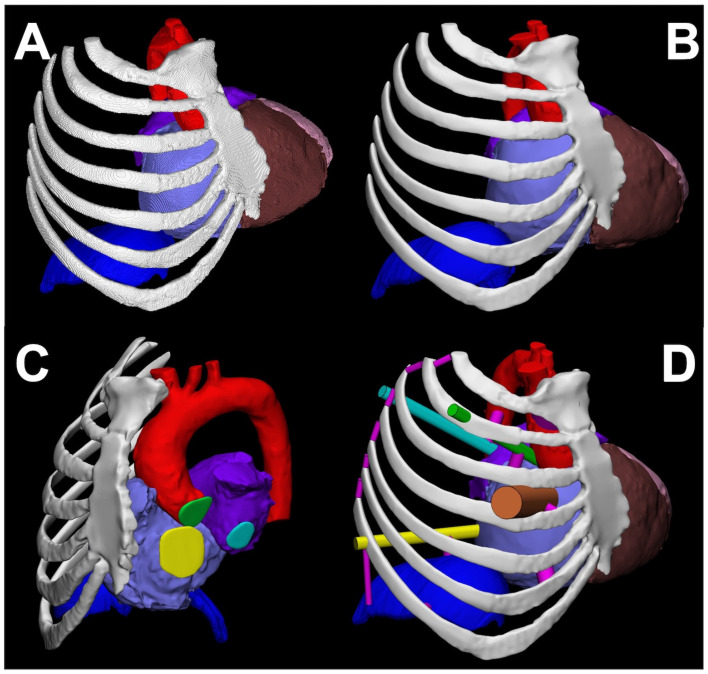
(**A**) The initially meshed digital model showing a rough surface due to wrapping of a mesh around the segmented voxel-based masks. The segmented structures include the ribs and sternum (white), the right hemidiaphragm (dark blue), the aorta (red), the right ventricle (brown), the right atrium (lavender), the left ventricle (pink), and the left atrium (purple). (**B**) The mesh in CAD showing a smooth surface after local and global smoothing operations. (**C**) The valve annulus planes (disks) are individually segmented and shown as they are the surgical targets. The plane of the aortic (green), tricuspid (yellow), and mitral (cyan) valves are shown. (**D**) Orthogonal cylinders are added to the valve planes to show their full volumetric orientation with respect to the working port, represented by a 25 mm diameter orange cylinder between ribs 3 and 4. After assessment of the valves, this working port was placed in the third intercostal space in consultation with the cardiothoracic surgeon because it uniquely provides access to all three valves. Connecting pins (magenta) are added to reinforce the model for successful 3D printing.

This step helps with the mesh optimization of the STL model and any geometrical modifications which are typically unique to each clinical case (Figure 4). Trimming any isolated elements or extraneous anatomy, spike removal, smoothing of the surface mesh file, labeling of the anatomical structure and patient medical record number, and wrapping a more uniform mesh are some of the tasks performed at this stage. Sometimes the STL conversion is suboptimal which can lead to issues of holes between triangular facets, inverted normals, and/or self-intersecting elements which can ultimately contribute to the non-manifoldness of the mesh. For 3D printing, the mesh must be manifold. Mesh editing software enables a mesh analysis and rectification of any errors. Furthermore, many research facilities and hospitals have CAD models verified by physicians (e.g., radiologists or surgeons) to confirm anatomical accuracy by overlaying the mesh file contours onto the source DICOM images. Any inaccuracies in anatomy are addressed by converting the mesh file back to a segmented mask and editing the mask. This is critical for conveying the correct information to the interventionalists for their pre-procedural planning. CAD also enables adding support structures for reinforcing the model and making it monolithic for 3D printing. The finished model can be exported as an STL or in another format such as 3MF which includes color and texture information.

### 2.4. File Preparation

**Figure 5:**
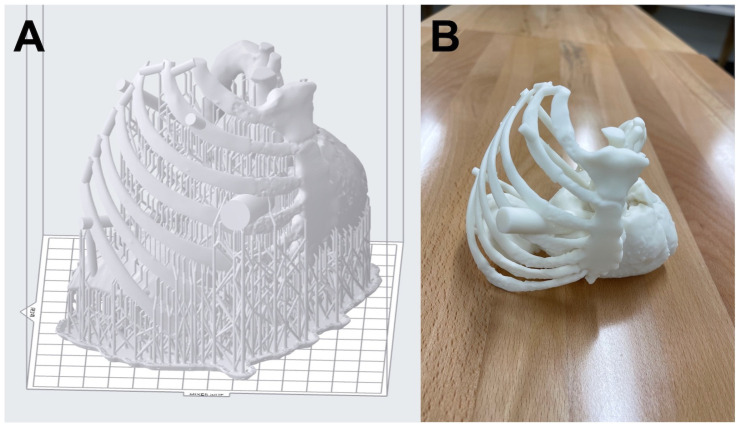
(**A**) Print preview of the 50% scaled model with support structures. The model needed to be scaled to 50% of its original size to fit onto the build platform of the desktop inverted VP 3D printer. (**B**) The final model 3D printed in White resin after post-processing.

The file is ready to be imported into the slicing software. Slicing software creates step-by-step or line-by-line instructions to create the final print on a 3D printer [24]. Many VP printers come with licensed software that guide the user with steps including the addition of support structures to ensure successful 3D printing as shown in Figure 5A. Nevertheless, it is important to create an internal Standard Operating Procedure (SOP) to minimize, if not eliminate, errors in printing by following an established protocol regardless of the operator 3D printing the anatomic models. Individuals should be trained to operate not just the 3D printer, but the software as well. Some errors that get introduced during this process are rescaled models, incorrect layer thickness, poor resolution of the print, insufficient support structures, and suboptimal orientation of the part [25,26]. Carefully scrutinizing the sliced preview, prior to 3D printing, can save vast amounts of time and resources, as this allows for the identification and prevention of avoidable errors and/or any potential print failures at an early stage. The printability checks in the slicing software are a great guide to ensure that models are adequately supported and that there are no other issues such as suction cups or floating elements etc.

### 2.5. Three-Dimensional Printing

The next step involves the actual 3D printing of the model. The printing technology to be used is a discussion between the ordering clinician, 3D printing engineer, and supervising radiologist. The clinician must understand the capabilities, limitations, and costs associated with the different 3D printing technologies and the engineer must understand the medical challenges that can be best addressed using the most appropriate technology. Desktop VP printers available in the current market are user-friendly and self-guiding, unlike earlier versions. Formlabs, Stratasys, Xometry, and Flashforge are a few companies that either manufacture desktop VP 3D printers or provide VP 3D printing as a service. Formlabs (Somerville, MA, USA) transformed the desktop VP 3D printing industry with their state-of-the-art Form series 3D printers which are now widely used in laboratories, industries, healthcare facilities, and research institutions. Material selection and controls are very easy with the new desktop VP printer. The sliced model or file is sent to the 3D printer either via ethernet or loaded using a USB drive, and the printer commences 3D printing in a layer-by-layer fashion.

As discussed earlier, the materials used for VAT polymerization involve photopolymer resins that can undergo photochemical reactions to solidify when exposed to specific wavelengths of light. Manufacturers add proprietary additives and components to achieve desired properties and characteristics [27,28]. Most standard photocurable resins offer a balance of properties suitable for a wide range of applications providing excellent resolution, strength, and surface finish. Standard resins are widely used for functional prototyping and concept modeling. Engineering resins that have enhanced mechanical properties provide higher strength and durability, making them apt for functional and durable parts production. Flexible and elastic types of photocurable resins are commonly used to make soft-tissue components including vasculature, skin, or muscles during anatomical 3D printing. It is important to note that the availability and specific formulations of resins can vary depending on the manufacturer and the type of VP technology used. Most 3D printer manufacturers provide a range of resin options with different properties to cater to various applications.

### 2.6. Post-Processing

Typically, all the 3D printed parts go through post-processing to varying degrees, and VP 3D printing is no different. The VP printed parts are carefully removed from the build table and rinsed thoroughly with Isopropyl Alcohol (IPA) to remove excessive resin from the part. After the rinse, the part is placed inside a UV curing chamber for post-curing. The post-curing helps fully set the part properties and achieve the maximum attainable part strength. It is important to not over-cure the part which makes it brittle whereas under-curing may leave toxic resin behind and result in a weaker part [29]. The 3D printed model of the endoscopic triple cardiac valve repair case is shown in Figure 5B after post-processing. Following the manufacturer’s instructions is critical during post-processing. Sterilization is the next step for the parts that will come in contact with patients [30] or that need to be taken into the sterile field within the operating room. Printed VP models of surgical guides and implants go through steam, ethylene oxide, or gamma radiation sterilization [31]. Care should be taken while sterilizing to avoid introducing unwanted errors such as inaccuracies due to warping and melting through heat. In the medical setup, SOPs should be developed, tested thoroughly, and followed. The settings and parameters should be carefully documented in the SOPs to avoid any potential deviation from the procedure which could introduce significant errors.

### 2.7. Final Inspection

**Figure 6:**
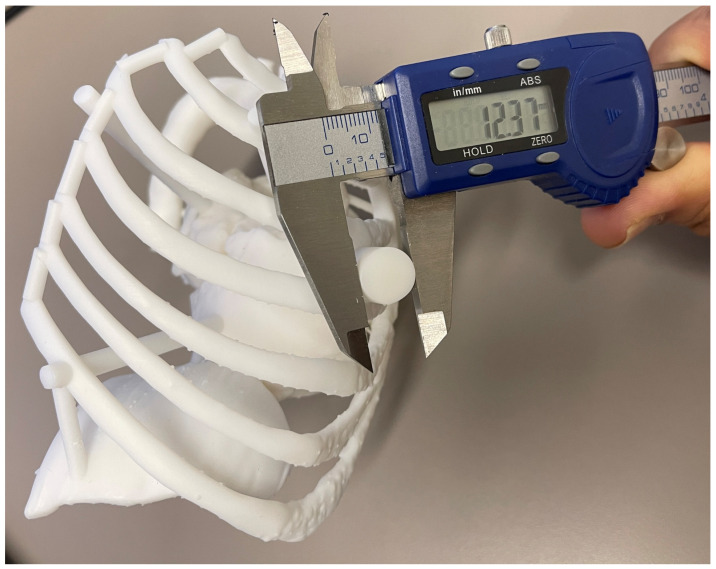
The working port cylinder diameter is measured with digital calipers to assess the accuracy of the entire 3D printed model. The designed cylinder was 12.5 mm (at 50% scale), and therefore, the measured diameter (12.37 mm) is 0.13 mm lower (1% deviation) and within acceptable limits.

Using FDA-approved segmentation software in combination with validated 3D printers and materials is an excellent preemptive strategy to maintain the quality of 3D printed anatomical models. It is highly recommended to first perform benchmark 3D printing with all the new 3D printers as well as third-party materials before fabricating any patient-specific models. A good sanity check is to print a reference cube (20 mm edge length) along with every patient-specific model and measure the cube to ensure that the 3D printing process was consistent and that there were no unexpected issues. Further, reference features such as landing blocks or cylinders (Figure 6) can be designed in CAD at pre-defined locations within the anatomic models for subsequent measurement and comparison against the design.

## 3. Principles of Desktop Inverted VP 3D Printing

**Figure 7:**
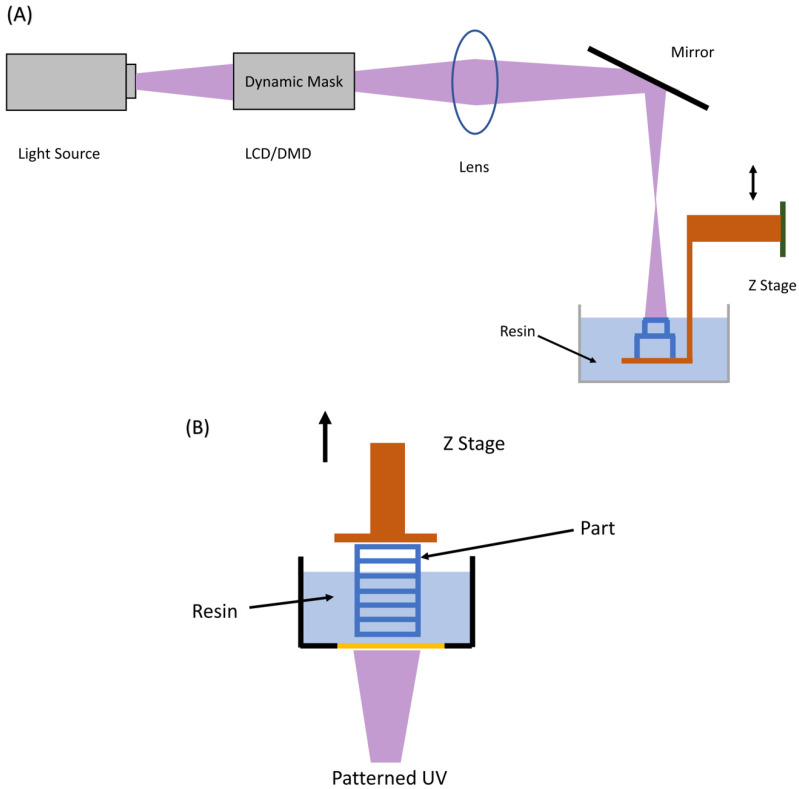
(**A**) Top-Down VP with the critical components highlighted. (**B**) Bottom-up VP with the important components labeled. The transparent membrane is shown in yellow at the bottom of the resin reservoir [32].

VP 3D printing (Figure 7), invented in 1986 by Charles Hull, is one of the oldest, highly mature, and most widely used industrial 3D printing techniques. It can produce large-size models, higher-resolution objects, and complex structures with much more accuracy compared to the MEX 3D printing technique. VP operates by utilizing a UV laser that is programmed to focus on a specific position within a container of photopolymer resin. This type of resin is responsive to UV light and will solidify and form highly interconnected networks when exposed to it, effectively creating a thin layer of the intended 3D object. The platform is subsequently adjusted upwards to permit the solidification or printing of the next layer. This solidification process is repeated to stack thin layers to form the final 3D printed object. Finally, the parts are washed with solvents to remove uncured material from the printed object. VP 3D printing is further subdivided into three methods based on the components and printing technique.

### 3.1. Platform Motion Based

As the name suggests, in this technique, the platform is moved while printing/stacking different layers. There are two ways in which the platform can be moved, i.e., upward or downward. Hence, this technique is further sub-classified into two components:

#### 3.1.1. Top-Down Approach

In the top-down approach (Figure 7A), the UV is projected from the top and the platform is moved vertically downwards as the layers are solidified, meaning that the resin is present on top of the movable build platform. At first, the build platform is situated near the upper part of the reservoir, causing only a slim layer of resin to be uncovered on the surface. This layer is then transformed into a solid state by the laser. Next, the build platform moves downwards, and a roller evenly distributes a fresh layer of uncured resin. To ensure a robust bond between the layers, the depth of the cure should surpass the thickness of the resin layer, allowing the newly treated layer to stick to the previous one(s). The light source (Figure 7A) can either be a laser (SLA, spot exposure), an array of UV LEDs (area exposure) masked by a liquid crystal display (LCD) which is also called masked SLA (MSLA), or a digital light projector (DLP, area exposure) manipulated by a dynamic mirror device (DMD).

#### 3.1.2. Bottom-Up Approach

In the bottom-up approach (Figure 7B) the UV light (laser/LED/DLP) is projected from the bottom and the platform is moved vertically upwards as the layers are solidified. The build platform is located close to the bottom of the reservoir initially, leaving a thin layer of liquid resin sandwiched between the build plate and the transparent window. This thin layer is then subjected to UV light, and subsequently, the build platform is raised to let liquid resin refill the space between the platform and the reservoir. This process is repeated step by step until the entire part is constructed. This technique generates smoother parts as the liquid resin makes full contact with the smooth bottom surface of the reservoir, resulting in a more even layer of resin. It is also safer because the laser remains inside the device, ensuring that the operator is not inadvertently exposed to harmful radiation. Furthermore, the curing process is carried out in a closed environment thus preventing oxygen exposure and inhibition during the photopolymerization process. The refill process occurs naturally through gravity, negating the need for recoating using a roller.

### 3.2. Laser Motion Based

This technique uses the motion of the laser instead of the platform to print thin layers, and the platform remains stationary. This technique can be further classified into two types: projection-based stereolithography (PSL) and scanning-based stereolithography (SSL). PSL can print an entire layer in a single shot of laser exposure which usually happens via the generation of patterned laser lights; however, SSL, on the other hand, scans the surface (like platform motion-based) of every individual layer to create those patterns. PSL produces higher resolution parts which are typically of small size, limited by the size of the patterned laser light. SSL, on the contrary, can be employed for large-size printing but suffers from a lower resolution. The printing speed of PSL is higher than SSL because the whole layer is fabricated in a single instance of exposure.

### 3.3. Continuous Liquid Interface Production (CLIP)

**Figure 8:**
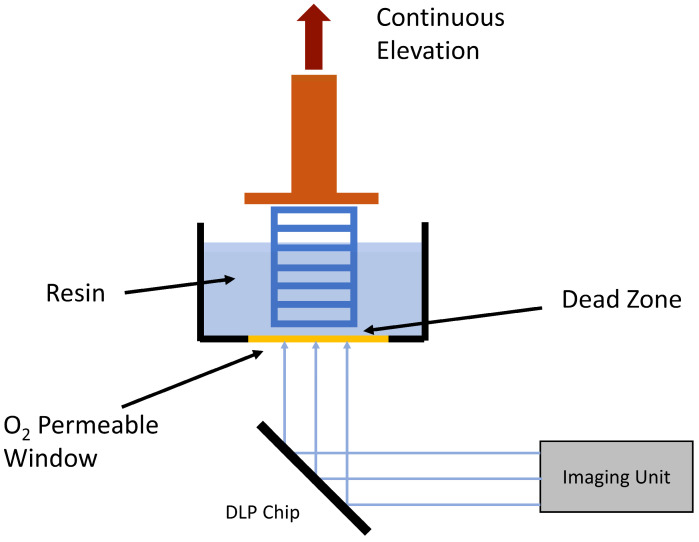
CLIP technology enables continuous fabrication of the model, unlike layer-by-layer approaches. This is achieved by creating a “dead-zone” at the window surface using oxygen-based inhibition of the radical chain photopolymerization reaction [32].

It is one of the latest innovations in VP 3D printing which significantly improves the post-print layer peel operation [11]. The post-print layer separation is a time-consuming process and CLIP technology overcomes that by creating a dead zone of liquid resin between the cured resin and oxygen-permeable membrane, allowing continuous curing of resin (Figure 8).

## 4. Advantages of Desktop Inverted VP 3D Printing for Medicine

**Figure 9:**
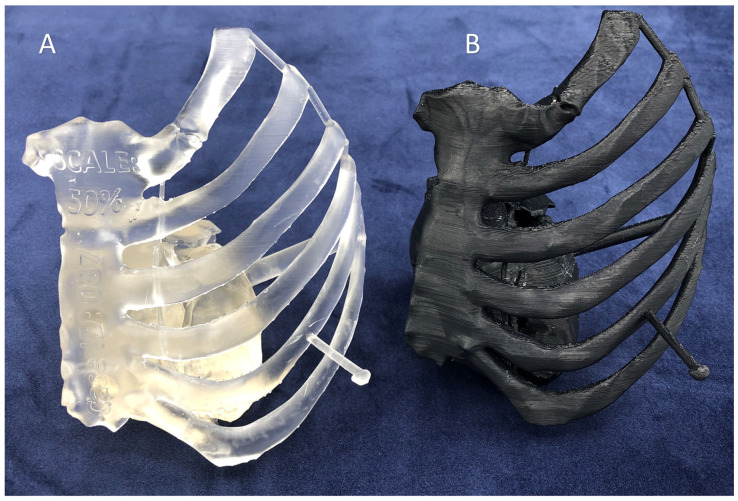
Comparison of the surface quality of a 3D printed model for planning minimally invasive coronary artery bypass grafting fabricated using Inverted VP 3D printing (**A**) on the Formlabs Form3B printer and (**B**) Material Extrusion (MEX) 3D printing on the Stratasys F120 3D printer. The layers are barely visible in the VP-printed model, but clearly visible in the MEX-printed model. There are also typical surface irregularities introduced by MEX printing.

**Figure 10:**
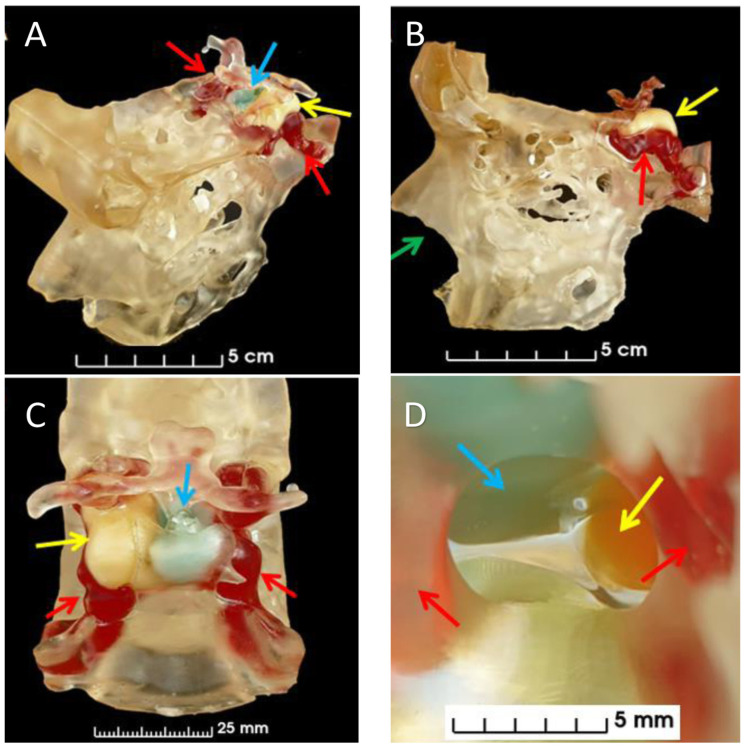
A 3D printed model was created for a patient with a pituitary tumor to assist in surgical planning and enhance patient comprehension. Different structures, including the pituitary gland, pituitary adenoma, carotid arteries, and bone, were separated and printed in varying colors by adding colored resin to the transparent structures. Multiple views of the model were presented, including an angled view (**A**), lateral view (**B**), posterior view (**C**), and view from the nose (**D**). The pituitary gland (blue arrows), adenoma (yellow arrows), carotid arteries (red arrows), and the site for surgeon access to the pituitary gland through the pituitary sella (green arrows) [34].

The very first 3D printing process invented was top-down VP in the 1980s [33]. The inspiration for the inventor Charles Hull came from tabletop coatings hardened using Ultraviolet (UV) radiation. For over two decades, the top-down VP systems were bulky and expensive systems that were not easily accessible to most users. With the expiration of several key patents in the 2000s, VP 3D printing was optimized for wider accessibility. One of the revolutionary changes was inverting the technology to a bottom-up approach from the original top-down design. This drastically reduced the size and cost of the technology, thereby successfully bringing it into the desktop realm (Table 1). Because VP 3D printing uses liquid photopolymers for the additive manufacture of models, the surface quality obtained is the best compared to other 3D printing technologies that utilize powder or filament as feedstock (Figure 9). The visual appearance and surface quality of medical models are a vital component of their utility, and inverted VP 3D printing is well suited for the fabrication of such medical models. Inverted VP 3D printing can fabricate anatomic models using transparent materials that enable powerful visualization of the internal anatomic features/landmarks. By incorporating colored pigments in the photopolymeric resins, or 3D printing hollow models using a clear resin to be later filled in with colored resins, models can be fabricated in a multitude of colors (Figure 10) [34]. A smoother surface promotes better sterilization for surgical guides and implantable devices. The smooth surface, particularly at a low layer thickness of 0.025–0.05 mm, offers a superior aesthetic feel to the models compared to other technologies that suffer from rough surfaces due to the filament or powder-based nature of the feedstock. Inverted VP 3D printing can capture intricate features such as vasculature (Figure 9) as long as the supports and orientation are meticulously planned, whereas this can be a challenge with MJT due to the requirement of high pressure washing and with MEX due to the necessity of soluble supports that are typically only available with more industrial 3D printing systems. The surface area of the model in direct contact with support structures is very low in desktop inverted VP 3D printing due to the fine control over the polymerization process and the relative strength of the support structures since they are fabricated from the same material as the model.

**Figure 11:**
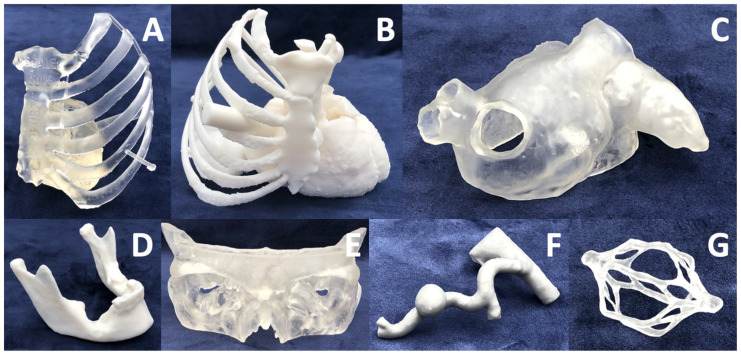
Sampling of the anatomical models fabricated at the University of Cincinnati Department of Radiology 3D Printing Laboratory. (**A**) Minimally invasive coronary artery bypass planning, (**B**) Endoscopic triple (mitral/tricuspid/aortic) valve planning, (**C**) Left atrial appendage occlusion device sizing, (**D**) Mandibular osteomyelitis surgery planning, (**E**) Orbital floor surgery planning, (**F**) Hepatic artery pseudoaneurysm intervention planning, and (**G**) Complex inferior vena cava filter retrieval interventional planning. Note that models are not shown to scale.

The accuracy of VP 3D printing is also the highest amongst 3D printing technologies, second to only MJT. For applications such as surgical guides, this accuracy is important to ensure an accurate fit. Medical models typically contain complex geometries and organic shapes since they are generally based on patient-specific anatomy (Figure 11). Inverted VP can fabricate complex geometries so long as the orientation and support structure placement is carefully planned. The inverted VP 3D printers are much more affordable compared to their top-down industrial counterparts due to their reduced form factor as well as the relatively low volume of resin required in the vat for 3D printing. For most medical models, the build volume of these desktop-inverted VP machines is enough for fabrication. The liquid photopolymeric nature of materials affords superior versatility because the base photopolymer can be combined with a multitude of materials. For instance, resins can be created for diverse applications such as dental crowns, engineering prototypes, anatomic models, surgical guides, jewelry castings, flexible components, etc., and fabricated on the same desktop inverted VP 3D printing system. Because single materials with well-known properties are utilized, they are better suited for biocompatibility compared to other technologies that use multiple materials for model, color, and supports. The inverted nature of the process enables a modular design such that the vat can be easily swapped out for different materials with minimal effort. Due to the availability of a plethora of inverted VP 3D printers and materials, the competitive landscape has resulted in a relatively low cost of consumables. Depending on the usage, the annual cost of consumables can be higher than the initial investment to procure a desktop inverted VP 3D printing system.

## 5. Medical Applications of Desktop Inverted VP 3D Printing

### 5.1. Anatomic Models

Desktop inverted VP 3D printing has been used for 3D printing diverse anatomical models with a sufficient level of accuracy for diagnostic use [35,36]. Twenty-one models of fetal cardiovascular anatomies spanning a broad range of diagnoses including tetralogy of Fallot, hypoplastic left heart syndrome, dextrocardia, double outlet right ventricle, and atrioventricular septal defect were 3D printed from postmortem microfocus CT data using a Formlabs Form 2 printer [37]. The models were printed in 1:1 scale using rigid resin as well as with 5-fold magnification. Three-dimensional printed models fabricated using a Formlabs Form 1+ printer helped re-interpret mesenteric vascular anatomy for planning colon cancer resection [38]. Virtual reality and desktop VP 3D printed models were found to be similarly valuable, but advantageous compared to conventional visualization in a study focused on congenital heart disease [39]. A Formlabs Form 2 printer was used to fabricate models with internal nasal anatomy to plan the repair/simulate cerebrospinal fluid leak [40,41]. A transconjunctival excision of an orbital conjunctival cyst was planned in a dog using a 3D printed model of the cystic lesion, a portion of the skull including the lacrimal, maxillary, zygomatic bones, and their respective foramina fabricated using a Formlabs Form 2 printer [42]. An apical muscular ventricular septal defect closure was planned using an elastic model 3D printed using a Formlabs Form 3 printer [43]. The elastic 3D printed model was sutured as part of the trial run. An anatomic model and implant were both fabricated using a Formlabs Form 2 3D printer for one-step resection of a meningioma and for cranial reconstruction [44]. Using the same 3D printer and freeware, a bone prosthesis of the frontal process (zygomatic bone), the zygomatic process (temporal bone), and a portion of the parietal bone were fabricated for craniofacial reconstruction [45]. The femur (right distal) and proximal tibia were segmented, and 3D printed using a Formlabs Form 2 3D printer for planning revision ligament (anterior cruciate) reconstruction [46]. The anatomy comprising the femoral artery, vein, pelvis, and cuboid mold encasing and supporting the structures for ballistic gel soft tissue casting was 3D printed using a Formlabs Form 2 3D printer [47]. Using the same printer, solid 3D printed models of the middle cerebral artery aneurysms were obtained and used to develop wax casts and hollow silicone models [48]. A patient-specific model of congenital scoliosis secondary to an L3 hemivertebra was fabricated and used for preprocedural planning [49]. A model of the femoral head and acetabulum was obtained for planning revision hip surgery [50].

Models of coronary anomalies for eight patients were created using a Form 2 printer for evaluation purposes [51]. A 3D printed model of a double-outlet right ventricle having multiple ventricular septal defects was created for surgical decision-making [52]. The models included 1:1 and 2:1 sized blood pools and a separate model of the myocardium. Hollow aneurysm models were 3D printed using a Form 2 printer for developing a modular in vitro neurovascular simulation tool for the rehearsal and planning of intracranial aneurysm embolization [53]. Models of an aortic valve, left atrial appendage, and normal/diseased mitral valve were created using transesophageal echocardiographic (TEE) images to establish an open-source methodology for the generation of 3D anatomic models from routine TEE datasets [54]. A Formlabs Form 1+ 3D printer was used to print flexible aortic root models for developing transcatheter aortic root repair technologies [55]. A Formlabs Form 2 printer was used to 3D print patient-specific nasal replicas for visualization and optimization of personalized irrigation strategies [56]. Patient-specific temporal bones were 3D printed for surgical simulation and evaluation while ensuring operability, low-cost, and high resolution [57]. The volatile organic compounds generated from the drilling of temporal bones 3D printed using a Form 2 printer using their standard white resin were found to be within applicable Occupational Safety and Health Administration levels [58]. An aortic root model was 3D printed using a Form 2 3D printer to aid in the training and simulation for transcatheter aortic valve replacement [59]. A complex truncus arteriosus model consisting of the patient’s heart and main vessels was 3D printed using a Form 2 printer and found to improve multidisciplinary communication [60]. Low-cost models were 3D printed for training endovascular aneurysm repair using a Form 1+ printer and flexible resin and found to reduce procedure time [61].

At the University of Cincinnati Radiology 3D Printing Laboratory, over 300 anatomic models have been 3D printed for planning various interventions and surgeries in the cardiovascular, oromaxillofacial, and abdominal specialties, among others (Figure 11). Most of these models were 3D printed using desktop inverted VP technology. The models were 3D printed using transparent (Figure 11A,C,E,G), opaque (white, Figure 11B,D, grey, Figure 11F), and elastic (Figure 11C) resins. The cost of materials alone was around $25–50 per model; however, the total cost of 3D printing a model is around $2200–2750 when a comprehensive cost assessment was conducted over one full year of operations [5,62]. The estimates when the models were outsourced to industry are similar. On average, the models saved about 30 min in the operating room which translates to a theoretical cost savings of $2900 per patient. The models also demonstrated high clinical utility based on feedback from the requesting proceduralists. The diverse application of inverted VP 3D printing for fabricating anatomic models for pre-surgical planning and simulation demonstrates the applicability and utility of this technology in medicine.

### 5.2. Surgical Guides and Surgical Planning

**Figure 12:**
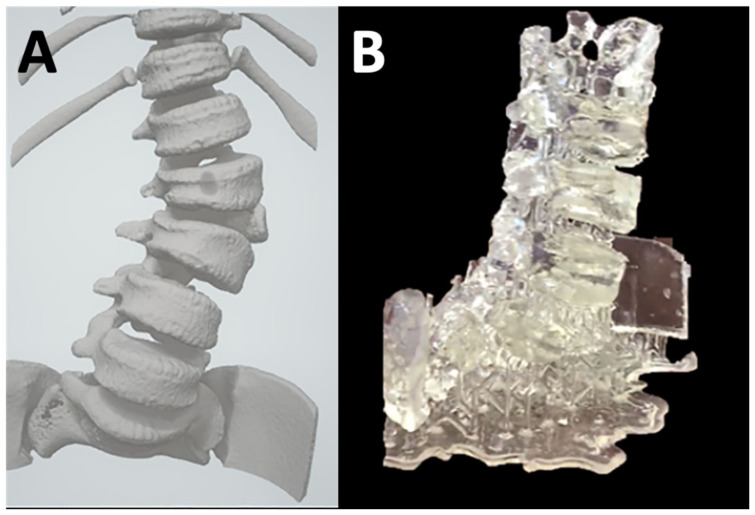
(**A**) Anterior view of the virtual 3D model for Lumbar scoliosis demonstrating a fully segmented L3 hemivertebra for a pediatric patient. (**B**) The resulting 3D printed model aided in the surgical planning. The patient subsequently underwent a successful L3 hemivertebra resection and L2–L4 posterior fusion. Pediatric spinal deformity surgeries are challenging procedures requiring significant expertise and resources. The 3D printed model enabled more precise and efficient surgical planning compared to 2D radiological images. The model also assisted patient families in intuitively visualizing the anatomical abnormalities and as a result were useful during consultations [49].

The following review includes the publications where desktop inverted VP 3D printing was used for fabricating surgical guides, which are one of the prime applications that have accelerated at a great pace in the last decade. The applications have ranged from developing surgical guides to placing pedicle screws for spinal surgeries to cranioplasty applications. Dental guides are also very common 3D printed parts fabricated using VP. VP 3D printing has become an important tool in fabricating surgical guides for complex spinal surgery, including for pediatric patients with skeletal dysplasia [63,64] as well as for simply 3D printing just the models for informing patients and guiding surgeons [49] (Figure 12).

The complexity associated with certain surgeries is alleviated using 3D printing technology. To solve a complex issue of leakage of cerebrospinal fluid (CSF) due to prolactinomas, a preoperative surgical model was fabricated to support the surgery on a skull defect, and it was reported that the 3D model improved the understanding of the multiple CSF leakage site and supported surgical planning [40]. Three-dimensional printed models instead of digital models have helped surgeons determine the middle colic artery (MCA) bifurcation position compared to anatomical landmarks and to assess the trajectory of accessory MCA when operating colon cancer [38], as well as for surgical planning of a large chest wall skeletal defect where guides were manufactured using VP resin (Dental Surgical Guide (SG) resin), and 3D printed on a Formlabs Form 2 printer [65]. In a case study of a 12-month-old child with a double-outlet right ventricle and two ventricular septal defects, medical professionals utilized 3D printed models in addition to the available imaging data [52]. The printed models provided additional insight, leading the clinicians to conclude that the arterial switch operation was not a feasible option. The 3D models were instrumental in determining that a biventricular repair was preferable to a univentricular repair, and ultimately, the surgical approach showed improvement thanks to the use of the models. Due to the complex anatomy, transcatheter aortic root repairs (TARR) are not available, hence human-derived models can be useful before conducting animal testing. In an attempt to develop TARR models, four different resins were utilized and tested for validation, stress, and coronary tests. Results revealed that while all four roots printed with different resins were within the dimensional tolerances, Visijet M3 crystal resin was too rigid for the transcatheter device, while although flexible, photopolymer gel SUP705 and Formlabs flexible resin broke during nominal balloon inflation. HeartPrint resin root adapted to the catheter device resulted in no valve blow out. Hence, 3D printed TARR technologies can replicate the human anatomy as well as guarantee physiologic coronary flow [55].

**Figure 13:**
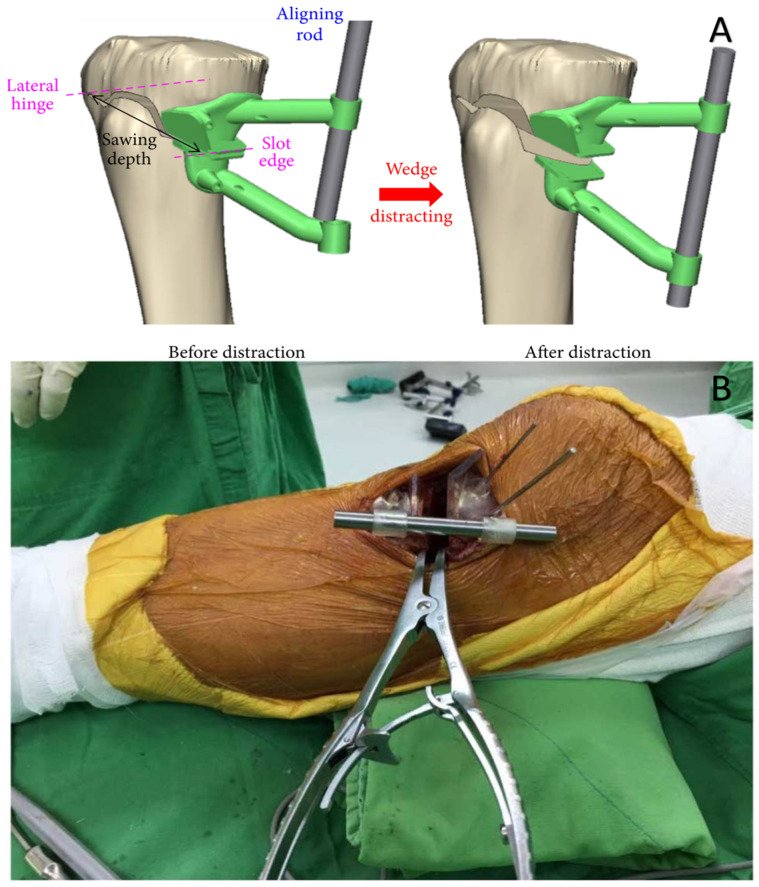
Application of 3D printing in medial open wedge High Tibial Osteotomy. It is a technique to correct varus deformity due to knee arthritis by making a precise cut in the upper Tibia. The success of this technically demanding procedure depends on restoration of the alignment of the lower limb in the coronal plane. This necessitates significant blood loss and radiation exposure. (**A**) Pre-operative planning where virtual surgical modeling is used to conceptualize a novel osteotomy and distraction guide. It incorporates a cutting guide for the precise 3-dimensional osteotomy. The inner surface of the guide is planned to intimately conform to the native Tibial anatomy, resulting in quick and precise placement on the proximal medial Tibia. For fixation of the guide, holes are planned to allow usage of smooth pins. (**B**) Intra-operative photograph demonstrating the 3D printed surgical guide fixed to the proximal tibia with smooth pins. A lamina spreader has distracted the osteotomy to ensure that the mechanical axis of the lower limb and the tibial slope is accurately corrected [68].

Time and cost are important factors for medical applications. In various surgical applications such as the surgical repair of isolated orbital structures [66], cranioplasty [67], and patient-specific instruments for medical opening wedge high tibial osteotomy [68] (shown in Figure 13), VP printing is proving to be cost-effective and results in reduced operative time. With the digitization of the surgical planning process, many resection and implant surgeries have become a single-step process unlike conventional techniques or two-step procedures [69], further assisting in time reduction. Reductions in overall surgery time and cost have been reported when surgical guides or anatomical models have been utilized for spinal muscular atrophy disease [70], and using in-house Virtual Surgical Planning (VSP) for maxillofacial reconstructive procedures [71]. A novel technique that can be used for in-house 3D printed patient-specific glenoid guides reported significantly lower inclination deviation and substantially reduced time compared to commercial guides [72]. A pre-operative CT scan-based clinical strategy was proposed, which used 3D printed spinal screw trajectory guides (3D-SSTG), reducing the radiation burden on the patients and the related time and cost. This was the first case where the 3D-SSTG printed on the Formlabs Form 3B with “Surgical Guide Resin” has been used in the clinical strategy for Morquio Syndrome, while previous applications were mostly focused on the thoraco-lumber pedicle screw placement in adults [63]. A digital workflow of a technique to fabricate interim fully removable dental prosthesis using in-office 3D printers was proposed which validate the clinical studies for VP 3D printing and their cost-effectiveness [73]. The 3D printed models used in percutaneous interventions in congenital heart disease for simulation and planning of catheter-based procedures have been demonstrated to reduce complications, and operating room times, as well as limit exposure to radiation [74].

A good manufacturing technique needs support from the standpoint of the availability of the appropriate material. When it comes to desktop VP printing, usable materials and their biocompatibility are playing a major role in advancing the technology. Researchers have focused on evaluating monomer release content from resin [75] and biodegradable polymers for Additive Manufacturing (AM) applications focusing on the biomedical field including collagen, gelatin, fibrin, chitosan, alginate, cellulose, hyaluronic acid, and polyhydroxyalkanoates with applications ranging in exoskeleton devices and exoprostheses, surgical implants, tissue engineering, 3D tissue modeling, integrated organ-on-a-chip, and drug release [76]. VP 3D printing has helped in improving the overall safety of a procedure where surgical guides were employed to place screws in the exact location out maneuvering complex anatomy. In such an attempt, a group of researchers virtually created a “resection line” object through CT scan data to guide resection during surgery for a patient with intraosseous meningioma and from the same object created a patient-specific implant using a VP 3D printer [44]. Accurate guide-wire placement is critical for successful femoral neck stabilization surgery, but current methods for guide-wire placement can be challenging and imprecise [74]. Researchers have proposed an approach to produce custom drill guides rapidly that can enhance the accuracy of guide wire placement while mitigating the risk of mispositioning during surgery [77].

Many surgeries require operating on varying architectures of bony structures making them difficult and time-consuming; training beforehand can be a great tool to reduce any associated risk. The case of the skull base middle cranial fossa surgical approach to the internal auditory canal (IAC) is of such a kind. To ease the surgical planning and improve the surgical simulation, researchers used VP to 3D print the MCF where the IAC was filled with yellow resin. The surgeons rated the drilling process of the model at 9.2 out of 10 indicating that the 3D printed model is a favorable tool to create a realistic representation of the anatomy [78]. In a skull base surgery, 3D printed models were useful in locating the meningioma and the bilateral vasculature first in the model suggesting the benefits of VP in resident training and patient education [79]. Desktop VP has impacted canine surgeries as well. A research report describes the surgical planning and transconjunctival excision of a conjunctival cyst in a dog using a 3D printed anatomic model. A portion of the dog skull was 3D printed using 300µm layer thickness on the Formlabs Form 2 3D printer. Surgery was simulated using the 3D model printed which demonstrated that a transconjunctival approach to the cyst was likely feasible without necessitating an orbitotomy. This surgical technique permitted a minimally invasive procedure that eliminated the need to perform an orbitotomy or sacrifice the globe [42].

The capability of several 3D-printable materials which can imitate the infiltration characteristics of human tissue can be used for training purposes where challenging vascular anatomy is desirable [80]. Additionally, a study was conducted to determine whether incorporating 3D printed models with CT scans improved the agreement between clinicians and the surgical approach when assessing femoral and tibial tunnels. While the addition of 3D printed models did not significantly impact agreement for attending orthopedic physicians, it did lead to a higher agreement rate (76%) for fellow physicians compared to CT images alone (63%) (*p* = 0.050). Overall, fellow physicians had a higher agreement rate (74%) with the 3D printed models than with CT alone (65%) (*p* = 0.049) [46]. Clinicians and researchers have found that 3D printed models of coronary artery anomalies developed from CT data are useful in visualizing coronary artery anatomy and their abnormalities and complement viewing 3D CT scans [51]. These models can aid in planning both surgical and minimally invasive procedures, including the diagnosis and treatment of airway diseases. Surgeons can process anatomic information more quickly than with images, helping them choose the optimal surgical approach and ultimately resulting in shorter surgery times and reduced complications [81].

#### Accuracy of Surgical Guides

High accuracy is needed for 3D printed surgical guides because of the tight fit they must achieve on the targeted anatomy for the planned cut or other action. In contrast, although the accuracy of 3D printed anatomic models is also important, it is generally not as critical as that of anatomic guides because of their diagnostic nature. An accuracy of 1mm or better maybe generally acceptable for most anatomical models fabricated for diagnostic and planning purposes. The accuracy of the printed guides [64] and implants has been verified by many researchers [82]. While the virtual planning software allows to collect data for creating accurate implants or surgical guides [83], comparing the actual implants with planned implants allows the assessment of their accuracy [82]. The effect of build orientation and layer height on the marginal fit and the internal gap of models have been evaluated reporting that the 45 and 60 degree build orientations gave clinically acceptable models in line with milling and cast restoration, while the layer height of 100 µm and 50 µm had a similar marginal fit when printed on the D2-120 (Hephzibah, Incheon, Korea) DLP 3D printer [84]. Even in canine surgeries and other veterinary applications, the 3D printed guides fabricated using desktop VP 3D printing have proven to be accurate. Thirty-two cervical pedicle screws in three large breed dogs using 3D printed drill guides resulted in 90% of the screws getting planted without any vertebral breach indicating the technique to be superior [85].

Moreover, there is also a great interest in evaluating the impact of sterilization on the accuracy of surgical guides and implants. Sterilization is a necessary step for the safety of patients and the steam heat or dry heat used during the process could affect the dimensional accuracy and the resistance. No significant difference has been reported for surgical guides made from poly methyl methacrylate (PMMA) when the pre-sterilized surgical guides were compared against the post-sterilized guides subjected to 120 °C in an autoclave [86]. The results could be attributed to the fact that the sterilization temperature was lower than the degradation temperature of PMMA. Table 2 reports the accuracy of various surgical guides and implants.

Since VP is often compared to other 3D printing technologies such as MJT and MEX, it is therefore important to compare the accuracy between these technologies. The error in printing is comparable between VP and MJT while the errors in MEX are almost double, although the cost of printing with VP is much less as compared to MJT [92]. When comparing the accuracy of PolyJet, VP, and MJT, the mean absolute difference (and the mean relative difference) between the 3D printed model and CAD are found to be 0.06 ± 0.05 mm (0.46 ± 0.51%) for PolyJet, 0.31 ± 0.33 mm (1.11 ± 0.70%) for MJT, and 0.09 ± 0.05 mm (0.66 ± 0.62%) for VP. It can be concluded that both the PolyJet and VP 3D printers met the required accuracy threshold for clinical applications [93]. In implant dentistry, various research has shown that the mean deviation of implants placed using guided surgery techniques is within acceptable tolerances, clearly demonstrating the efficacy of this technology [94]. Furthermore, when the accuracy of commercially available 3D printers for the production of surgical guides is compared, for small extent surgical guides, the mean error has been reported lowest for the Rapid Shape D40 with Sheraprint SG 100, followed by the Form 2 with Dental SG resin, the Cara Print 4.0 with biocompatible resin Dima Print Guide, the Stratasys J750 with VeroWhitePlus photosensitive resin, the Prodways P1000 with polyamide powder PA12-L 1600, and the Raise 3D Pro 2 with PLA filament. However, for large-extent surgical guides, the mean error has been reported lowest for Form 2, followed by the Cara Print 4.0, the Rapid Shape D40, the Stratasys J750, the Prodways P1000, and the Raise 3D Pro 2 [95].

### 5.3. Prosthesis and Hearing Aids

**Figure 14:**
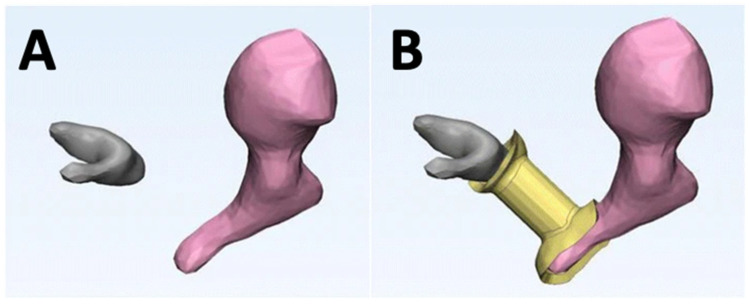
Surgical rebuilding of the ossicular chain to treat conductive hearing loss requires precise and patient-specific prosthesis (i.e., incus) (**A**) Rendered 3D model of the malleus (in grey) and the stapes (in pink) from CT after removal of the incus. (**B**) The in-situ design of the incus (in yellow) to create a unique prosthesis from CT scanner data [98].

**Figure 15:**
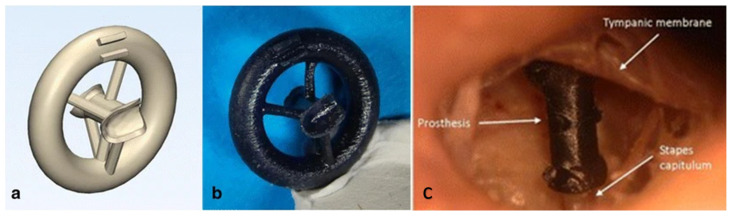
Formlabs Form 2 VP 3D printing technology was utilized to fabricate removed incuses from cadaveric human temporal bones. Four otologic surgeons were able to pair individual prostheses to the right parent bone, indicating that the VP technique can create a unique prosthesis from CT scanner data with high accuracy. (**a**) An STL model of the prosthesis where a circular structure with spokes was designed to support the printing of an incus. (**b**) 3D printed model of the prosthesis (i.e., incus) with support structure. (**c**) Prosthesis placed in the left middle ear between the manubrium and capitulum [98].

While 3D printing of life-size human models through 3D printing might seem a stretch [96], the past decade or so has seen huge success in the advancement of AM in hearing aids and prosthetics. Among the success of AM, VP has shown potential, with the ability to fabricate hearing aids loaded with drugs to prevent ear infections [97]. In the case of prostheses, they are unique in geometry and size, and improperly sized prostheses are one of the reasons for component failures. A study used Formlabs Form 2 VP 3D printing technology to fabricate removed incuses from cadaveric human temporal bones. Four otologic surgeons were able to successfully match each prosthesis to the correct parent bone, indicating that the VP technique can create a unique prosthesis from CT scanner data with high accuracy. This study addressed the well-known issue of creating accurate custom prostheses and suggests that the VP technique can be a valuable tool for creating patient-specific implants. (shown in Figure 14 and Figure 15) [98]. In utilizing desktop VP for hearing aids, some researchers have focused on delivering drugs locally through 3D printed and custom hearing aids [97]. When it comes to industry, the reports by Christian Sandstrom suggest that although non-disruptive, 3D printing usage for hearing aids is increasing steadily as more and more manufacturers are overcoming the challenges of training staff, and VP is one technology that is widely adopted by the big manufacturers of hearing aids [99,100]. However, the technology needs to overcome the clinical, financial, and technological barriers to enable much wider adoption [101].

### 5.4. Other Devices

VP also facilitates the fabrication of other major medical devices which can be utilized at the PoC. The importance of access to medical devices in low-resource or health crisis settings was highlighted by designing and producing an otoscope (a medical device used to examine the ear canal and eardrum) [102]. An innovative approach to designing and producing airway stents using mathematical surface functions and 3D printing technology that are tailored to individual patients was developed to help patients with various respiratory conditions breathe more easily [103]. A technique that uses 3D printing to create individualized orthopedic casts was introduced as an alternative to traditional plaster casts, which can cause discomfort and complications such as pressure sores and skin infections [104]. A new approach for sampling exhaled breath was developed using a 3D printed mouthpiece adapter to make it more comfortable and practical for patients [105].

**Figure 16:**
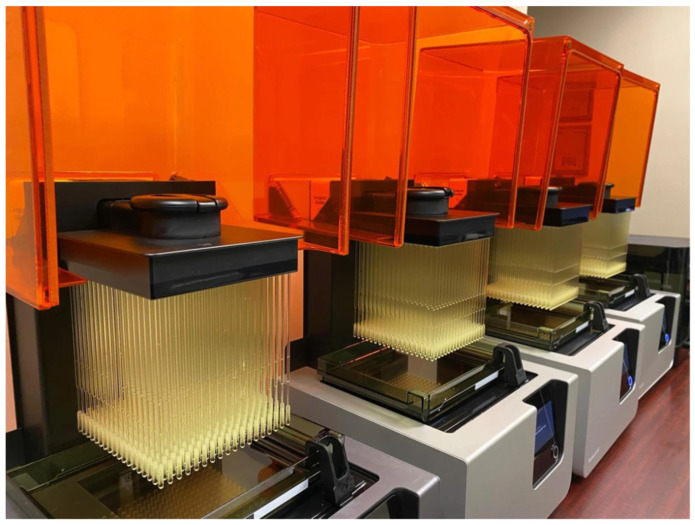
4 × 324 nasal swabs fabricated using desktop inverted VP 3D printing ready for post-processing. The swabs were 3D printed using SG resin at 0.1 mm layer height. They were then placed in pouches and sterilized via steam [106].

**Figure 17:**
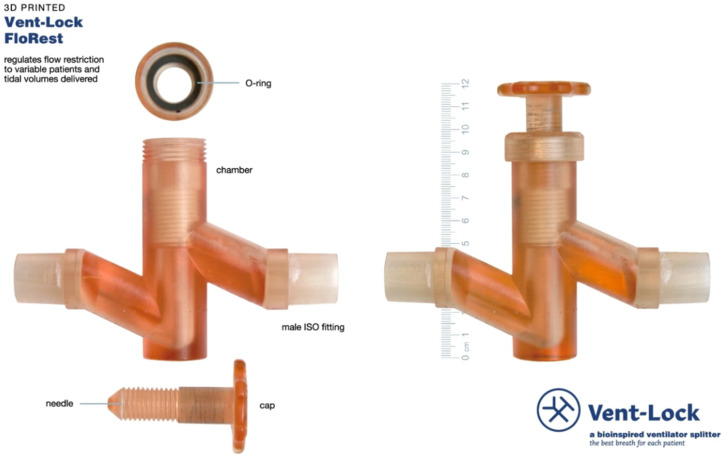
The Vent-Lock flow restrictor fabricated using desktop inverted VP 3D printing at 0.05 mm layer height using Surgical Guide (SG) resin. The flow restrictors were sterilized by dry vacuum autoclave [107].

Another 3D printing application is producing nasopharyngeal swabs for COVID-19 testing that can be customized to individual patients and quickly scaled up for mass production (Figure 16) [106]. Many medical devices are not designed with the needs of low-resource settings in mind and traditional manufacturing methods can be too expensive or too slow to meet the needs of these settings. For example, the development and production of Vent-Lock, a 3D printed ventilator multiplexer that allows multiple patients to be ventilated using a single ventilator (Figure 17), was developed as an example to address the need for rapid iteration and customization of medical devices during emergencies or pandemics [107]. Computational fluid dynamics simulations were also employed to evaluate the performance of 3D printed ventilator splitters and restrictors for multi-patient ventilation during the pandemic crisis [108].

Accurate guide-wire placement is critical for successful femoral neck stabilization surgery, but current methods for guide-wire placement can be challenging and imprecise. An approach has been proposed to produce custom drill guides that can improve the accuracy of guide wire placement during femoral neck stabilization surgery. The use of 3D printing technology can enable rapid customization of drill guides for individual patients and has been shown to improve accuracy and reduce the risk of mispositioning during surgery [77]. A miniature force sensor using desktop inverted VP 3D printing was developed which can be inserted into living tissue without causing significant damage, unlike current methods for measuring forces in living tissue that are often invasive and can cause tissue damage [109,110,111]. The authors also noted that the use of 3D printing technology can enable rapid and low-cost production of the force sensor, which could make it more accessible for researchers and clinicians. Numerous 3D printing techniques to fabricate intercranial vasculature have been reported including using desktop VP 3D printing [112]. A report describes the 3D printing of nanocomposite pills for drug delivery applications. Traditional manufacturing methods for producing pills are often limited by specialized tooling and the inability to easily produce complex geometries. The report proposes a new approach that uses desktop inverted VP 3D printing to produce pills with precise geometries and controlled drug release properties. The process of designing and producing the 3D printed pills is described, and the mechanical and drug release properties of the pills are evaluated in a series of in vitro experiments. It is demonstrated that the 3D printed pills have excellent mechanical strength and can be engineered to release drugs at a controlled rate. The use of nanocomposite materials in the 3D printing process can enable the production of pills with unique properties, such as increased bioavailability and enhanced drug delivery efficiency [113].

**Figure 18:**
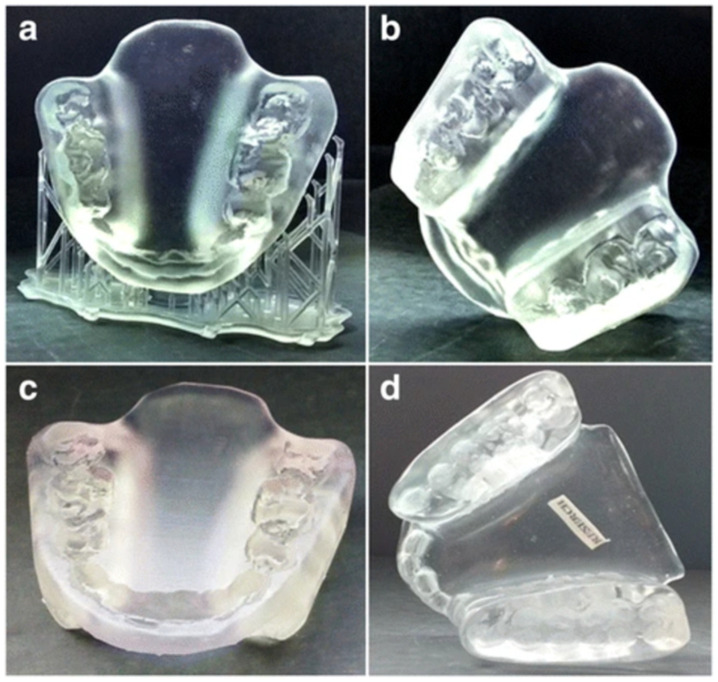
(**a**) The 3D printed oral stent immediately after 3D printing and (**b**–**d**) following support structure removal and post-processing. The stent was 3D printed using clear resin at 0.05 mm layer height. The authors did not use SG resin and the stent was not sterilized [114].

Researchers have developed customized oral stents (Figure 18) using VP technology which fit the patient’s anatomy, are comfortable for the patient to wear and reduce the time and cost associated with creating a custom oral stent compared to traditional methods. The stent was effective in immobilizing the patient’s head during radiotherapy treatment [114].

A summary of the important clinical applications of desktop inverted VP 3D printing is provided in Table 3. The applications span the gamut of anatomical regions and include anatomic models, guides, molds, devices (including drug-loaded), casts, adapters, and other devices.

## 6. Challenges of Desktop Inverted 3D Printing

**Figure 19:**
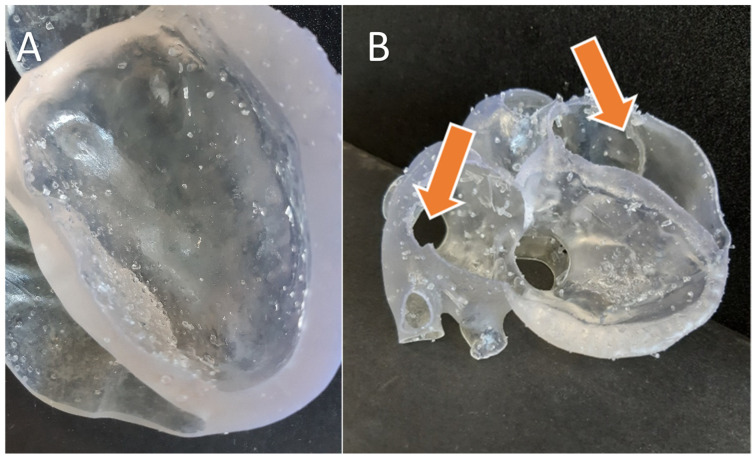
(**A**) Visible residual touch points from support structures post-removal in a 3D printed heart model (lower part) and (**B**) the upper part of the same heart model torn in some locations (arrows) due to softer material printing through VP. The model was 3D printed using flexible resin 100-micron layer height [121].

The development of technology is highly associated with constituent materials, capabilities, standardization, and ease of use. Desktop VP technology generally fails to produce parts faster and there are no specific standards that exist which is further compounded by the different subtypes of VP technologies that exist today. The desktop VP technology still needs to address the issues of overall print size, print time, efficiency, limitations in processing highly viscous materials, and capabilities in printing colored models [115]. Volumetric resin-based 3D printing promises to address several of the limitations of desktop VP 3D printing [116,117]. Companies like Xolo3D and Readily3D have developed commercial volumetric 3D printers, but the part size is currently limited to around an inch or a bit lower. VP can produce an extremely smooth surface finish model; however, it is limited to single-color and single-material prints. However, one strategy to overcome this limitation is to print clear models that are hollow and then introduce and cure the colored resin in the hollow spaces, although this strategy substantially increases the effort required and is applicable to only a subset of all potential applications [34]. On the other hand, MJT can print several colors and material properties in the same model, with a slightly rougher finish [82]. Full-color 3D printing can be powerful in the clinical domain to help communicate complex information in a more intuitive format. Some negative features brought forth by researchers include the time required to print models as well as the skilled training required for the equipment. While most researchers in the clinical world suggest reduced time in overall surgery due to surgical planning using VP or other rapid prototyping techniques, the actual cost of these events is still case-dependent and remains hidden due to limited use. On top of that, training requirements for the technology demand additional costs [15]. A comparison of the clinical performance of 3D printed surgical guides with the specific application to mandibular surgery and temporomandibular joint implants was performed [103]. The author concludes that although AM is cost-effective, the data published on the application of AM to mandibular surgery are often incomplete in relevant clinical outcomes and hence a full comparison with other conventional manufacturing techniques is not possible [118]. Some researchers have raised concerns about the limited availability of biocompatible materials that can be processed using VP and utilized for widespread production since some applications or devices need to come in close contact with the human body [105,119]. Furthermore, printing with VP technology requires well-defined support structures which can lead to print failures if not defined properly unlike PBF, and in the case of flexible material, the removed supports can leave behind visible marks (Figure 19) [120,121]. Additionally, the 3D printing of flexible models takes 2–3x longer compared to similar rigid models. Some other limitations include the lack of standardization [122] for clinical applications in terms of accuracy, verification of the printed product, and tooling. Overall, as the technology and the applications continue to grow, more efforts in standardization and improvement in the technology and materials are required. Although groups like the ASTM and photopolymer additive manufacturing alliance (PAMA) are working to develop standards, much progress remains to be achieved.

## 7. Conclusions

VP 3D printing is a versatile, high-quality, and accessible technology for use in healthcare settings to manufacture patient-specific models, guides, and a plethora of other devices. Although the technology has limitations such as the lack of ability to 3D print in full color, the inherent toxicity of base materials, and the liquid photopolymeric nature of feedstock, the excellent surface finish, accuracy, relatively low cost, ease of post-processing, and material versatility have facilitated the adoption of this technology within hospitals, medical device companies, and other research laboratories. The use of desktop printing has mitigated the cost and space constraints so that the technology is more amenable for hospital-based 3D printing. With the recent advent of volumetric 3D printing promising the super-fast fabrication of models without support structures and the continually expanding applications, the future of desktop VP 3D printing appears highly positive.

## Figures and Tables

**Table 1:** Qualitative overview of the major 3D printing technologies used in medical 3D printing.

Characteristic	Inverted VP	MJT	PBF	MEX	BJT
Surface Quality	High	High	Medium	Low	Low
Accuracy	High	High	High	Low	Low
Geometric Complexity	Medium	Medium	High	Low	High
Cost	Low	High	High	Low	High
Material Versatility	High	Low	Low	Medium	Low

**Table 2:** Accuracy of different surgical guides and implants.

Surgical Guide/Anatomical Area/Description	3D Printer and Resin	Accuracy Results	Ref.
Measure distance and angular deviations in faciolingual and mesiodistal locations of the implants	Form 2; Dental SG Resin	Placed and planned implants in mesiodistal location had a mean difference of 0.28mm while the faciolingual direction had 0.49mm, and the angulation deviations were 0.84° and 3.37°.	[82]
Effect of process parameters on the internal gap and marginal fit of model for dental implants	DLP printer D2-120; Polymethyl methacrylate (PMMA) resin	45° and 60° build orientations gave clinically acceptable models in line with milling and cast restoration processes while the layer height of 100 µm and 50 µm had similar marginal fit.	[83]
Compared milled surgical guides with 3D printed guides or printing device, resin material, and preoperative sterilization	Rapidshape D20II and Form2; NextDent SG resin	The location of the implant was influenced by both the type of printer used and the resin material at both the crest and apex. Despite these variables affecting implant position, the 3D printed guides were comparable to the milled ones and the displacement of the printed implants fell within an acceptable range for safety.	[87]
A drill guide for posterior atlantoaxial pedicle screw fixation	Form 1+; Acrylic resin	No difference between the actual and planned trajectories of axis and atlas of those screws	[64]
Surgical guides for cervical pedicle (CPS) screws in breed dogs	Form 2; Dental SG resin	Results showed 29 of 32 CPS were placed without any vertebral canal breach	[85]
Effect of sterilization on the stability and the accuracy of surgical guides	Form 2 and Simplant; Poly methyl methacrylate (PMMA) resin	No significant difference between pre-sterilized and post sterilized guides	[86]
Tooth, bone, and mucosa-supported surgical guides for linear and angular deviation	Custom VP machine; Stereocol resin	Surgical guides supported by tooth were more accurate as compared to other two (bone and mucosa) based on angular deviation of 2.91° ± 1.3°, 4.63° ± 2.6°, and 4.51° ± 2.1° (tooth, bone, and mucosa).	[88]
Evaluating accuracy of tooth supported SGs which were printed for various angle and arch.	Form 2; Dental SG	The build orientation angle had no significant effect on the guides	[89]
Impact of build angle on the material usage, accuracy, and speed	Form 2; Dental SG	The accuracy of the surgical template varied depending on the build angle, with 0-degree and 45-degree angles producing the most accurate templates while 90-degree angles produced the least accurate. The 0-degree build angle had the fastest printing speed, while the speed decreased with an increase in build angle, with 90-degree angles taking the longest time. However, the increased speed was at the expense of using more material, with the 0-degree angle using the most material and the 90-degree angle using the least amount of material.	[90]
Analyzed the precision of guides by the inserting reference screws mandibular models	3D Systems Viper; DSM Somos’ RX opaque resin	Reference screws can be positioned accurately using guides during guided bilateral sagittal split osteotomy	[91]

**Table 3:** Summary of the clinical applications of desktop VP 3D printing.

Human Anatomy or Application	Specific Applications	3D Printer and Resin	Title 4 Technology (SLA/DLP/CLIP)	Ref.
Spine	3D printed guide for spinal screw on C2 vertebra during operation	Form 3B, Surgical Guide resin	SLA	[63]
	A patient-specific model of congenital scoliosis secondary to an L3 hemivertebra (spine) was created for surgical planning	Form 2, Clear resin	SLA	[49]
	A drill guide for atlantoaxial pedicle screw positioning	Form1+, Acrylate resin (Somos 14120)	SLA	[64]
Skull	3D model was printed to understand the leakage of cerebrospinal fluid and surgical planning	Form 2, Grey resin	SLA	[40]
	A mold was 3D printed to give shape to the implant for orbital blow-out fracture	Form 2, Yellow resin	SLA	[66]
	Printed a mold using VP which was later used to create PMMA implant for cranioplasty	Form 2, Unknown	SLA	[67]
	Patient-specific models were printed to improve resident training and patient education for delicate carinal nerve structures	Form 2, Acrylic resin	SLA	[79]
	Anatomical model for resection of a tumor and cranioplasty	Form 2, Flexible resin	SLA	[44]
	Cranial bone prosthesis was 3D printed for feasibility study	Form 2, Gray v4 resin	SLA	[45]
	3D printed models of the middle cerebral artery aneurysms for creating wax casts	Form 2, White resin	SLA	[48]
	Patient-specific temporal bones were 3D printed	Form 2, White resin	SLA	[57]
Colon	3D printed model helped identify the bifurcation position for colic artery for colon cancer procedure	Form 1+ , White resin	SLA	[38]
Heart	Models helped with surgical planning in Multiple Ventricular Septal Defects and their relationship with aortas	Form 2, White resin	SLA	[52]
	Transcatheter aortic root repairs model was developed to replicate the coronary flow	Visijet M3, Crystal resin; Form 2, Flexible resin; Heart print, Clear resin	SLA, DLP	[55]
	3D printed heart models helped to enhance the understanding of coronary abnormalities.	Form 2, White resin	SLA	[51]
	Virtual Reality model was compared with VP model for congenital heart disease	Form 2, Flexible resin	SLA	[39]
	Model printed in planning of an apical muscular ventricular septal defect closure	Form 3, Flexible resin	SLA	[43]
	Models of an aortic valve, left atrial appendage, and normal/diseased mitral valve were printed	Form 2, Unknown	SLA	[54]
Airway	3D printing of patient-specific airway stents	Anycubic Photon Mono; Soybean-based biodegradable photopolymer resin	SLA	[103]
Aorta	Low-cost models for training endovascular aneurysm repair	Form 1+, Flexible resin	SLA	[61]
	An aortic root model for training simulation for transcatheter aortic valve replacement	Form 2, Grey resin	SLA	[59]
Hip	A model of the femoral head and acetabulum for revision hip surgery	Form 2, Unknown	SLA	[50]
	Femoral neck stabilization surgery	Form 3 using Grey V4 resin	SLA	[77]
Knee and femur	A patient-specific intraoperative guide was 3D printed for precise creation and distraction of high tibial osteotomy wedge	Form 2, Dental SG	SLA	[68]
	Physicians and surgeons evaluated whether the current femoral, as well as tibial tunnles, were adequate in revision anterior cruciate ligament reconstruction with and without the 3D models	Form 2, Acrylic resin	SLA	[46]
	3D printed the anatomy comprising the femoral artery, vein, and pelvis for training medical students	Form 2, Grey resin	SLA	[47]
Maxillofacial	When combined with virtual surgical planning, surgical guides for maxillofacial reconstruction improved the accuracy of bony reconstruction.	Form 2, Dental SG	SLA	[71]
	Middle cranial fossa filled with an internal auditory canal was printed to simulate realistic drilling process.	Form 2, white acrylic resin	SLA	[78]
	Tooth, bone, and mucosa-supported SG enhanced the accuracy of implants	Custom VP machine; Stereocol resin	DLP	[88]
	VP guides used to precisely insert screws on mandibular models	3D Systems Viper; RX opaque resin	SLA	[91]
Teeth	Tooth-relying VP guides improved the accuracy of implants.	Form 2; Dental SG Resin	SLA	[82]
Nose	Fabricated models with internal nasal anatomy to plan the repair/simulate a cerebrospinal fluid leak	Form 2, Grey acrylic resin	SLA	[40,41]
	3D print patient-specific nasal replicas for personalized irrigation strategies	Form 2, Acrylic resin	SLA	[56]
	3D printing of Nasal Swabs for COVID testing	Form 2 and Form 3B, standard FDA-approved resin	SLA	[106]
Ear	Printed prosthesis incuses of temporal bone to identify if VP can produce unique shapes to reconfigure ossicular continuity	Form 2, Black resin	SLA	[98]
	Drug-loaded hearing aid	Kudo3D Titan 2 HR 3D printer, Kudo3D 3DSR Flexible resin and hard resin mixed with drug	DLP	[97]
	Otoscope (a medical device used to examine the ear canal and eardrum)	Prusa i3 Pro-B with ABS Filament Mars Pro with Elegoo transparent resin	MEX/SLA	[102]
Pituitary gland	3D Models of pituitary tumors for surgical planning	EOS100 with Nylon PA2200, Stratasys J750, Prusa Research (Prague, Czech Republic) MK3 with PETG filaments, Prusa Research SL1 with transparent Resin	PBF/MJ/ MEX/VP	[34]
Mouth	Mouthpiece adapter to sample breath	Formlabs Form 3B, with Surgical Guide, Tough v5, and BioMed Clear resins	SLA	[105]
	Customized oral stent for head and neck radiotherapy	Form 2, standard clear resin	SLA	[114]
Other Device	A 3D printed ventilator multiplexer that allows multiple patients to be ventilated using a single ventilator	Form 2, Form 3, or Form 3B; SG resin	SLA	[107]
Bladder	Designing and producing a non-invasive miniature force sensor	Form3; Grey resin	Low Force Stereo- lithography (LFS)	[109]
Drug Delivery	Nanocomposite pills for drug delivery applications	Form 2; Resin made out of PEGDA, PEO, and the photoinitiator (TPO)	SLA	[113]

## Data Availability

No data was used for the research described in the article.
